# Hydroponic Treatment of *Nicotiana benthamiana* with Kifunensine Modifies the *N*-glycans of Recombinant Glycoprotein Antigens to Predominantly Man9 High-Mannose Type upon Transient Overexpression

**DOI:** 10.3389/fpls.2018.00062

**Published:** 2018-01-30

**Authors:** Sugata Roychowdhury, Young J. Oh, Hiroyuki Kajiura, Krystal T. Hamorsky, Kazuhito Fujiyama, Nobuyuki Matoba

**Affiliations:** ^1^James Graham Brown Cancer Center, University of Louisville School of Medicine, Louisville, KY, United States; ^2^The International Center for Biotechnology, Osaka University, Suita, Japan; ^3^Department of Medicine, University of Louisville School of Medicine, Louisville, KY, United States; ^4^Center for Predictive Medicine, University of Louisville School of Medicine, Louisville, KY, United States; ^5^Department of Pharmacology and Toxicology, University of Louisville School of Medicine, Louisville, KY, United States

**Keywords:** kifunensine, *N*-glycosylation, high-mannose-type glycan, mannosylation, subunit vaccine, *Nicotiana benthamiana*

## Abstract

*Nicotiana benthamiana* transient overexpression systems offer unique advantages for rapid and scalable biopharmaceuticals production, including high scalability and eukaryotic post-translational modifications such as *N*-glycosylation. High-mannose-type glycans (HMGs) of glycoprotein antigens have been implicated in the effectiveness of some subunit vaccines. In particular, Man_9_GlcNAc_2_ (Man9) has high binding affinity to mannose-specific C-type lectin receptors such as the mannose receptor and dendritic cell-specific intracellular adhesion molecule 3-grabbing non-integrin (DC-SIGN). Here, we investigated the effect of kifunensine, an α-mannosidase I inhibitor, supplemented in a hydroponic culture of *N. benthamiana* for the production of Man9-rich HMG glycoproteins, using *N*-glycosylated cholera toxin B subunit (gCTB) and human immunodeficiency virus gp120 that are tagged with a H/KDEL endoplasmic reticulum retention signal as model vaccine antigens. Biochemical analysis using anti-fucose and anti-xylose antibodies as well as Endo H and PNGase F digestion showed that kifunensine treatment effectively reduced plant-specific glycoforms while increasing HMGs in the *N*-glycan compositions of gCTB. Detailed glycan profiling revealed that plant-produced gp120 had a glycan profile bearing mostly HMGs regardless of kifunensine treatment. However, the gp120 produced under kifunensine-treatment conditions showed Man9 being the most prominent glycoform (64.5%), while the protein produced without kifunensine had a substantially lower Man9 composition (20.3%). Our results open up possibilities for efficient production of highly mannosylated recombinant vaccine antigens in plants.

## Introduction

Over the past decade, *Nicotiana benthamiana* expression systems using viral and non-viral vectors have become viable platforms for the production of recombinant proteins (Matoba et al., 2011; Whaley et al., 2011; Chen et al., 2013; Nandi et al., 2016). Taking advantage of the systems' capacity to rapidly express complex proteins within days, a number of novel biopharmaceutical proteins, including monoclonal antibodies and subunit vaccine antigens, have been produced in *N. benthamiana* and showed protective efficacy in preclinical animal challenge models (Santi et al., 2006; Massa et al., 2007; Mett et al., 2007, 2011; D'aoust et al., 2008; Lai et al., 2010, 2014; Landry et al., 2010; Wycoff et al., 2011; Karauzum et al., 2012; Chichester et al., 2013; Petukhova et al., 2013; Shoji et al., 2013; Garcia et al., 2014; Hiatt et al., 2014; Qiu et al., 2014; Mardanova et al., 2015; Pillet et al., 2015; Tsekoa et al., 2016). Medicago Inc. has recently obtained a U.S. Food and Drug Administration's emergency use authorization for *N. benthamiana*-expressed hemagglutinin-based virus-like particle vaccine for H5N1 influenza virus, and will soon initiate a multi-center Phase III clinical trial for a quadrivalent seasonal influenza vaccine, highlighting the regulatory and commercial feasibility of the plant expression technology for biopharmaceuticals development (http://medicago.com).

Subunit vaccines are composed of non-replicating/pathogenic microbial components containing critical epitopes and are therefore considerably safer than live-attenuated vaccines, but their inherently weak immunogenicity often poses challenges for sufficient vaccine efficacy (Schiller and Lowy, 2015; Vartak and Sucheck, 2016). One of the effective approaches to improve vaccine efficacy is targeting antigens to pattern recognition receptors on dendritic cells (DCs), macrophages and other antigen presenting cells (Kumar et al., 2009; Takeuchi and Akira, 2010). Among others, antigen mannosylation has been proposed as a promising strategy because it increases antigen uptake by mannose-specific C-type lectin receptors such as DC-specific intracellular adhesion molecule 3-grabbing non-integrin (DC-SIGN) and mannose receptor (Lam et al., 2007; Al-Barwani et al., 2014; Sedaghat et al., 2014). Upon binding to C-type lectin receptors, glycosylated antigens are internalized and subsequently targeted for antigen delivery and stimulation of T cell responses (Apostolopoulos et al., 2013; Van Kooyk et al., 2013). Thus, development of an efficient recombinant production platform for mannosylated glycoprotein antigens may facilitate vaccine development. Especially, Man_9_GlcNAc_2_, a high-mannose-type glycan (HMG) with 9 mannosyl residues (Man9) has a higher binding affinity to DC-SIGN than other HMGs with fewer mannoses (Man5-8) and complex-type *N*-glycans (Feinberg et al., 2001, 2007; Van Liempt et al., 2006). Therefore, our ultimate goal is the production of Man9-rich glycoprotein vaccine antigens in plants.

Mannose trimming reactions from the precursor Glc_3_Man_9_GlcNAc_2_ occur in the early stages of the *N*-glycan processing pathway following the removal of terminal Glc residues, in which endoplasmic reticulum (ER)-type and Golgi α-mannosidase-I proteins are responsible for the initial step of mannose trimming (Liebminger et al., 2009; Strasser, 2016). Here, we attempted to establish an optimal kifunensine-treatment procedure in a hydroponic culture of *N. benthamiana* to obtain highly mannosylated, Man9-displaying recombinant vaccine antigens. Kifunensine is an α-mannosidase I inhibitor, which has been used in mammalian cell culture systems to modify the *N*-glycan profile of glycoproteins to be rich in Man9 HMGs (Elbein et al., 1990). However, its application and optimal conditions in whole-plant transient overexpression systems have not been reported. We used cholera toxin B subunit (CTB) and the envelope glycoprotein gp120 of human immunodeficiency virus type-1 (HIV-1), both containing a C-terminal H/KDEL ER retention signal, as model antigens in the present study. H/KDEL-tagged proteins were used in this study because, although the ER-retention strategy has been frequently used to produce recombinant proteins in plants, the signal usually brings about predominantly Man6-8 glycoforms and is sometimes leaky, resulting in heterologous glycan compositions with few Man9 glycans (Petruccelli et al., 2006; Matoba et al., 2009; Gomord et al., 2010; Loos et al., 2011; Triguero et al., 2011; Wang et al., 2013; Hamorsky et al., 2015). CTB is a potent mucosal immunogen used in the internationally licensed cholera vaccine Dukoral®. We have recently shown that CTB is *N*-glycosylated when expressed in *N. benthamiana* (Hamorsky et al., 2013b, 2015). The *N*-glycosylated CTB (gCTB) bound to cell-surface DC-SIGN in addition to GM1-ganglioside receptors, indicating that the glycosylated vaccine antigen may elicit additional immunomodulatory activity via several C-type lectin receptors (Matoba, 2015). Furthermore, preliminary results showed that gCTB's DC-SIGN-binding affinity could be significantly enhanced when the protein was produced under kifunensine treatment (Hamorsky et al., 2015), providing a basis for the present study and for the development of novel C-type lectin receptor-targeting vaccines. HIV-1 gp120, on the other hand, is a primary target of broadly neutralizing antibodies, thus constituting an important component of experimental HIV-1 vaccines (Zhou et al., 2007; Karlsson Hedestam et al., 2017; McElrath, 2017). Our results in the present work demonstrate that recombinant glycoproteins transiently expressed in *N. benthamiana* have predominantly Man9 HMGs upon hydroponically treating the plant with kifunensine after vector inoculation, providing a new method for the efficient production of highly mannosylated antigens for vaccine development.

## Materials and methods

### Vector construction, expression, and purification of gCTB and gp120 in *N. benthamiana*

Proteins were transiently overexpressed using the magnICON® deconstructed tobamovirus vector (pICH11599, ICON Genetics, Halle/Saale, Germany; Marillonnet et al., 2004). Vector construction and purification of gCTB, which contains a C-terminal KDEL sequence, were described previously (Hamorsky et al., 2015). The gp120 construct used in this study was derived from an *env* clone of the CCR5-using clade C virus DU156 (Genbank No. DQ411852). See Supplemental Methods for vector construction, expression, and purification of gp120. The purified protein was analyzed via sodium dodecyl sulfate polyacrylamide gel electrophoresis (SDS-PAGE) whereas its concentration was measured by the bicinchoninic acid (BCA) protein assay using HEK293 cell-produced gp120_DU156_ (Immune Technology Corp, New York, NY) as a reference control.

### Kifunensine, ascorbic acids treatments of hydroponically grown *N. benthamiana* for transient protein expression

**Figure 1:**
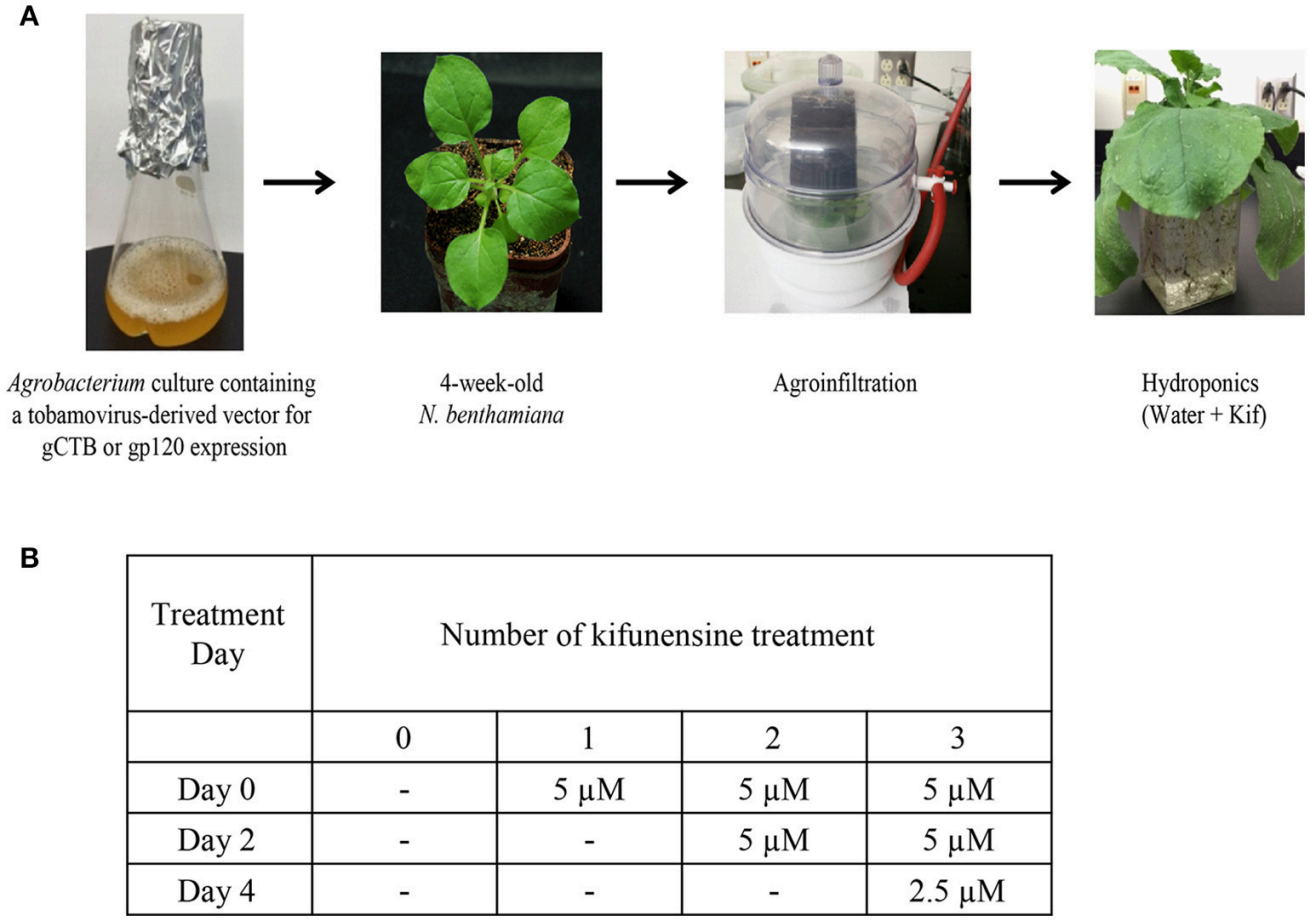
Study design and conditions. **(A)** A flow chart for agroinfiltration and hydroponic kifunensine treatment of *N. benthamiana*. **(B)** Kifunensine treatment conditions. The α-mannosidase I inhibitor was added at different time intervals; at the time of hydroponic setup (Day 0), and then added every other day, i.e., Day 2 and then Day 4 at the dose indicated.

Following agroinfiltration, 12 plants were removed from soil and transferred to hydroponic cultures for varying kifunensine (kif) treatments (Cayman Chemical, Ann Arbor, MI) with each group containing 3 plants, viz., control receiving no kif at all (0 kif), plants receiving kif only once (1 kif), twice (2 kif) and/or thrice (3 kif) during post inoculation growth (Figure 1). Protein extraction and purification was carried out at 5 days post inoculation (dpi) as described below. For ascorbic acids treatment, nine plants were used. Under the 3 kif conditions, a final concentration of 0.3 mM of l-ascorbic acids, adjusted to pH 5.8, was added to the hydroponic culture. RNA extraction and protein purification were performed at 2 and 5 dpi, respectively.

### RNA extraction, reverse transcription, RT-qPCR

Total RNA was extracted from 100 mg of fresh plant leaf material (*n* = 9). Plant tissues were lysed by grinding the tissue using liquid nitrogen and with a mortar and pestle. The samples were prepared by QIAShredder (Qiagen) and RNAqueous Phenol-free total RNA Isolation Kits (Thermo Fisher Scientific, Waltham, MA), following manufacturer's protocol. After total RNA isolation, TURBO DNA free kit (Thermo Fisher Scientific) was used to eliminate genomic DNA. For reverse transcription, first strand cDNA (2 μg) was synthesized using the High-Capacity cDNA Reverse Transcription Kit (Thermo Fisher Scientific). RT-qPCR was performed on an StepOnePlus™ Real-Time PCR System (Thermo Fisher Scientific) with SYBR Green PCR master mix (Thermo Fisher Scientific). The primers for *BiP, PDI*, and *bZIP60* and RT-qPCR conditions were followed as described previously (Hamorsky et al., 2015).

### Biochemical analysis of gCTB glycans

Endoglycosidase H (Endo H) and peptide-*N*-glycosidase F (PNGase F) digestions were performed as described previously (Matoba et al., 2009). Briefly, CTB proteins (2 μg) were incubated with Endo H or PNGase F (2000 units each) overnight at 37°C, separated by SDS-PAGE, transferred to a PVDF membrane, and probed with anti-CTB antibodies. Band intensities were measured using Carestream MI SE software. Data was expressed as a percentage of the proportion of glycosylated band remaining after each enzymatic digestion compared to untreated glycosylated band for a given kifunensine treatment. For plant-specific glycan detection, rabbit anti-xylose and anti-fucose antibodies (Agrisera, Vännäs, Sweden; 0.1 μg/mL and 0.05 μg/mL, respectively) were used as probes to detect gCTB in immunoblot analysis.

### Quantification of gCTB in *N. benthamiana* leaf extract

GM1-ganglioside-capture enzyme-linked immunosorbent assay (GM1-ELISA) was used for the detection and quantification of gCTB using a commercial CTB (List Biological Laboratories, Campbell, CA), as described previously (Matoba et al., 2009; Hamorsky et al., 2013b).

### High performance liquid chromatography-mass spectrometry analysis of gp120 *N*-glycans

Glycan profiling was performed as previously described (Matoba et al., 2009; Hamorsky et al., 2013b). For reference, recombinant gp120 produced in HEK cells (DU156.12, Clade C (Immunetech # It-001-RC1p) was used. Briefly, glycans were released from gp120 by hydrazinolysis, which were pyridylaminated and separated by reversed phase high-performance liquid chromatography (RP-HPLC) and size-fractionation (SF)-HPLC. The glycan structures were determined by RP-HPLC and tandem mass spectrometry (MS/MS), with their retention times and MS/MS profiles compared with those of an in-house MS/MS library constructed using commercial 2-aminopyridine (PA)-labeled standards of known isomeric configurations.

### Statistical analyses

Statistical significance was analyzed by one-way ANOVA with Bonferroni's multiple comparison test, using the GraphPad Prism 6.0 software. Differences were considered statistically significant if *P* < 0.05.

## Results and discussion

**Figure 2:**
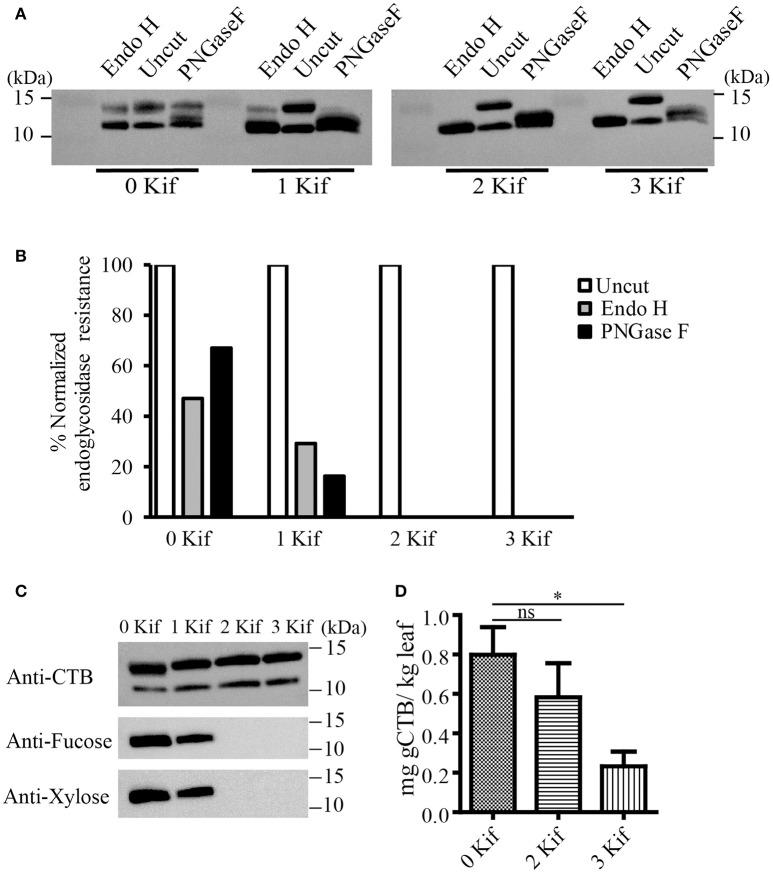
Endoglycosidase digestion of gCTB. **(A)** A representative immunoblot showing gCTB treated with a mock control (uncut), Endo H or PNGase F upon 0, 1, 2, and 3 kif treatments. **(B)** Densitometric analyses to calculate the fraction of glycosylated band resistant to either of the enzymes. Band intensity was normalized by calculating the fraction of residual undigested glycosylated gCTB band remaining after overnight digestion with respective glycosidases over glycosylated band intensity of an undigested gCTB sample, both receiving similar doses of kifunensine treatment. **(C)** A representative immunoblot showing gCTB from 0, 1, 2, and 3 kif conditions probed with α-fucose and β-xylose antibodies. **(D)** The quantification of gCTB_kif_ in *N. benthamiana* using GM1-ELISA from clarified extracts. Statistical significance was analyzed by one-way ANOVA followed by Bonferroni's multiple comparison tests [^*^P < 0.05; ns, not significant (*P* > 0.05)].

We set up a series of hydroponic cultures with each group receiving different doses of kifunensine during the period following agro-infiltration to harvest (Figure 1A). For transient overexpression of gCTB in *N. benthamiana*, the magnICON tobamovirus replicon vector was employed, which was delivered via vacuum-mediated agroinfiltration (Hamorsky et al., 2015). Then plants were transferred from soil to water and treated with varying doses of kifunensine (Figure 1A). Water in the hydroponic cultures was changed every other day along with a fresh dose of kifunensine based on the treatment regimen (Figure 1B). Groups of plants were treated without (termed 0 kif) or with kifunensine either once, twice or thrice (termed 1 kif, 2 kif, and 3 kif, respectively). Kifunensine-treated groups received 5 μM at a time except the 3 kif group, which received 2.5 μM prior to the day of harvest (Figure 1B). To examine the effect of kifunensine treatment on *N*-glycan profiles of gCTB, the purified protein from different kifunensine treatment groups (gCTB_Kif_) was subjected to Endo H and PNGase F digestions. Endo H is a glycosidase which cleaves within the chitobiose core of high mannose and some hybrid oligosaccharides from *N*-linked glycoproteins. PNGase F, on the other hand, cleaves mammalian *N*-glycans (including HMGs) between the innermost *N*-acetylglucosamine (GlcNAc) and Asn residues but fails to cleave those containing α(1, 3)-linked fucose, which are mostly found in plant and some insect glycoproteins (Wilson et al., 2001; Bardor et al., 2003). Western blot analysis probed with a goat anti-CTB antiserum showed two distinct bands for gCTB without any glycosidase treatment (Figure 2A and Supplementary Figure 1, Uncut lanes). This is due to incomplete *N*-glycan occupancy at Asn4 of the protein; the upper band corresponds to glycosylated gCTB and the lower band indicates the aglycosylated form (Hamorsky et al., 2015). Despite that gCTB contained a KDEL C-terminal ER retention signal, the protein was recalcitrant to Endo H and PNGase F digestion (Figure 2A, 0 Kif lanes). This indicates that gCTB was not retained in the ER effectively and thus modified with complex and plant-specific glycoforms in the Golgi. Conversely, the results revealed the limitation of the KDEL tag-based ER-retention strategy to enrich HMGs on this protein. By contrast, the 2 kif condition appeared to be sufficient to modify the glycan composition of gCTB to a HMG-rich profile, because gCTB's glycans were almost entirely cleaved by Endo H and PNGase F in the 2 kif and 3 kif groups but not in the 0 and 1 kif groups (Figures 2A,B). Additionally, gCTB was no longer detectable by anti-fucose and anti-xylose antibodies after two rounds of kifunensine treatments, indicating that the levels of plant-specific glycans containing α(1, 3)-linked fucose and β(1, 2)-linked xylose moieties were significantly reduced compared to single and no-kifunensine treated conditions (Figure 2C). Thus, these results demonstrate that hydroponic kifunensine treatment of *N. benthamiana* is effective at reducing plant-specific glycoforms while increasing HMGs in gCTB's glycan profile under transient overexpression conditions.

**Figure 3:**
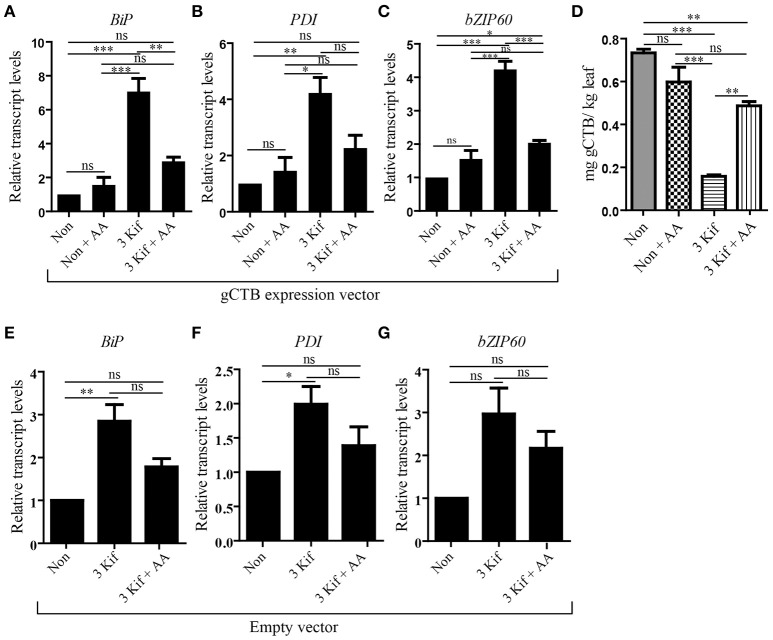
qRT-PCR analysis of ER stress related genes. **(A–C)** Total leaf RNA was isolated at 2 days post vector inoculation under no kifunensine treatment (Non), no kifunensine treatment with 0.3 mM ascorbic acids (Non + AA), 5 mM kinfunensine treatment (3 Kif) or under 5 mM kifunensine plus 0.3 mM ascorbic acids (3 Kif + AA). All groups have undergone the same procedural manipulations except for Kif or AA treatments. The expression levels of *BiP* **(A)**, *PDI* **(B)**, and *bZIP60* **(C)** were quantified by qRT-PCR. The 18S rRNA was used for the normalization of cDNA amount. Values indicated as fold increase to the average normalized value for the Non group and are expressed as means ± SEM of biological replicates (*n* = 9). **(D)** Quantification of gCTB from Non, Non + AA, 3 kif and 3 kif + AA conditions in *N. benthamiana* leaf extract using GM1-ELISA (*n* = 9). **(E–G)** qRT-PCR analysis of *BiP* **(E)**, *PDI* **(F)**, and *bZIP60* **(G)** of empty vector-inoculated plants. Plants were treated the same way as above, excluding the Non + AA conditions. Values indicated as fold increase to the average normalized value for the Non group and are expressed as means ± SEM of biological replicates (*n* = 9). Statistical significance was analyzed by one-way ANOVA followed by Bonferroni's multiple comparison tests [^*^P < 0.05; ^**^P < 0.01; ^***^P < 0.001; ns, not significant (*P* > 0.05)].

To test the impact of kifunensine treatment on gCTB accumulation, clarified leaf extracts were analyzed by GM1-ELISA. Results indicated that the gCTB yield was affected by kifunensine treatment; 2- and 3-kif conditions decreased the yield by ~30 and 75%, respectively (Figure 2D). To understand the mechanism for the reduction of gCTB accumulation under kifunensine treatment, we measured transcript levels of ER stress-related genes; previous studies showed that the modification of glycan structure might cause ER stress, which was associated with the reduction of a translation rate (Schneider et al., 1978; Lageix et al., 2008). ER stress induces the unfolded protein response (UPR). Basic-region leucine zipper 60 (bZIP60) is a transcription factor involved in a major arm of UPR in plants, which activates the expression of ER-resident molecular chaperons (Iwata and Koizumi, 2005; Hamorsky et al., 2015). Thus, we analyzed the expression of bZIP60 and two representative ER chaperons, luminal binding protein (BiP) and protein disulfide isomerase (PDI). Two days post vector inoculation under 5 mM kinfunesine supplemented conditions, we found that the expression levels of *BiP, PDI*, and *bZIP60* significantly increased by 7.0, 3.5, and 3.2 fold, respectively, compared with those of non-treated plants (Figures 3A–C). Collectively, these results suggest that kifunensine treatment induced strong ER stress, which in turn led to the reduction of gCTB expression levels. It is known that ER stress gives rise to reactive oxygen species (ROS), which causes the reduction of a translation rate mediated with the protein kinase GCN2 (Lageix et al., 2008; Liu et al., 2008). Based on this mechanism, we hypothesized that ascorbic acid might block ROS signaling and subsequent reduction of gCTB expression in kifunensine-treated *N. benthamiana*. To test this hypothesis, plants were incubated for 2 days under the co-treatments with 0.3 mM of ascorbic acid and 5 mM of kifunensine. As shown in Figures 3A–B, the ascorbic acid co-treatment significantly suppressed the kifunensine-induced elevation of *BiP, PDI*, and *bZIP60* transcript levels by ~50%, although they were still 2.9, 2.0, and 2.2-fold higher, respectively, than those of no-kifunensine conditions. Consistent with this, the reduction of gCTB yield associated with kifunensine treatment was significantly recovered by ascorbic acid co-treatment (though still ~35% lower than non-kifunensine treatment conditions) at 5 days post vector inoculation (Figure 3D). Meanwhile, ascorbic acid alone did not induce a significant change in ER stress marker gene expression or gCTB accumulation levels (Figures 3A–C). Higher doses of ascorbic acid were not effective at improving gCTB yield any further (data not shown). To further dissect the ER stress response, we analyzed the ER stress marker genes in plants that were infiltrated with an empty vector and treated under the same hydroponic conditions. The results were overall similar to those of gCTB-expressing plants; kifunensine treatment increased *BiP, PDI*, and *bZIP60* levels by 2–3 folds, while co-treatment with kifunensine and ascorbic acid did not show such effects (Figures 3E–G). Thus, it seems that kifunensine treatment alone induces significant ER stress in plants, at least under the hydroponic conditions employed here, although overexpression of recombinant proteins could exacerbate the stress further. Taken together, these results demonstrate that, although hydroponic kifunensine treatment of *N. benthamiana* causes ER stress and thereby reduces gCTB yields upon transient over-expression, ascorbic acid co-treatment can mitigate the adverse effect and recover the recombinant protein expression levels.

**Figure 4:**
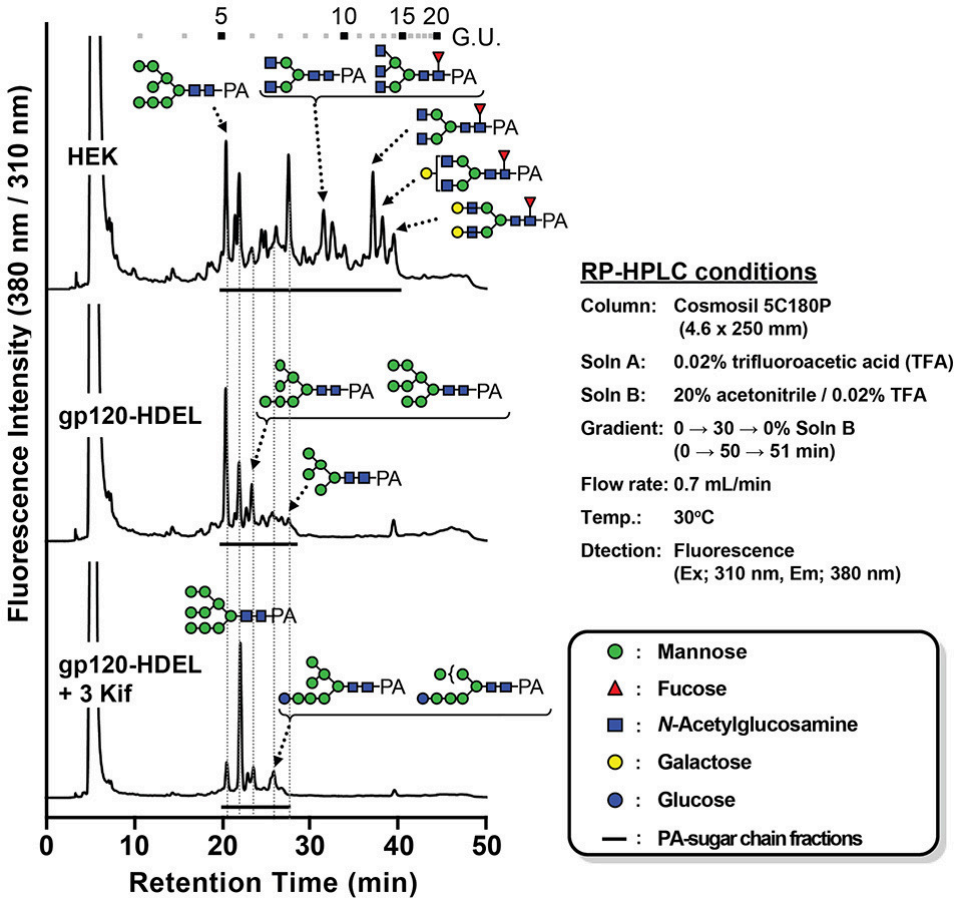
*N*-linked glycan profile of HIV-1 gp120. Chromatogram showing MS analyses of RP-HPLC separated pyridylamino (PA)-labeled glycans isolated from different growth conditions along with sugar legends (HEK: HEK 293T cell line; gp120-HDEL: plant-produced recombinant gp120-HDEL).

Next, we evaluated the impact of *N. benthamiana* kifunensine treatment on the *N*-glycans of recombinant HIV-1 gp120. As gp120 is one of the most heavily *N*-glycosylated viral proteins known so far (Kwong et al., 1998; Zhou et al., 2007), the protein provides an extreme case example to demonstrate the effectiveness of the present method. Additionally, it has been shown that the *N*-glycans of gp120 on primary HIV-1 isolates are predominantly HMGs (Doores et al., 2010; Bonomelli et al., 2011). Thus, the development of a high-mannose-rich recombinant gp120 is deemed important for an effective HIV vaccine. Since gp120 has a large number (15–25) of *N*-glycans (Kwong et al., 1998; Zhou et al., 2007), we employed 3 kif conditions described above (see Figure 1B). The recombinant HIV-1 gp120 from the clade C strain DU156 was expressed using the magnICON vector. As observed in gCTB expression (Figure 3), gp120-expressing plants showed a significant increase in ER stress marker gene expression under kifunensine treatment conditions, but the stress response could be blunted by ascorbic acid co-treatment (Supplementary Figure 2). The plant-produced gp120 was purified using a 3-step purification procedure including immobilized metal affinity chromatography followed by *Galanthus nivalis* lectin and a final diethylaminoethyl (DEAE)-based ion-exchange chromatography. The lectins of *G. nivalis* bind to D-mannose and have been used for the purification of HIV gp120 (Srivastava et al., 2002; Martin et al., 2008). In SDS-PAGE analysis, the plant-expressed gp120 showed a noticeably smaller molecular weight (~75 kDa) than human embryonic kidney (HEK293T) cell-produced gp120 (~120 kDa) (Supplementary Figure 3A). This could be due to differences in their glycosylation patterns, including compositions and occupancy; the theoretical molecular size of the plant-expressed gp120 without glycans is 52.9 kDa based on its amino acid composition. Glycans account for approximately half of the molecular mass of gp120 (Behrens and Crispin, 2017; Ward and Wilson, 2017). Thus, some, if not most, of the potential *N*-glycosylation sites may not have been glycosylated in plants. Nevertheless, the plant-made gp120 showed a similar binding curve to that of the mammalian cell-produced counterpart in a sandwich ELISA using the broadly neutralizing, anti-CD4 binding site monoclonal antibody VRC01 (Wu et al., 2010; Hamorsky et al., 2013a) and an anti-gp120 antiserum, suggesting that the plant-produced protein, overall, retains antigenic integrity of gp120 (Supplementary Figure 3B). To dissect the glycan profile of gp120, a combination of comparative high-performance liquid chromatography (HPLC) and mass spectrometry (MS) was carried out. Results indicated that gp120 from HEK293T cells contained a mixture of terminal mannose (39.4%) and/or β(1, 4)-galactose-linked (18.4%), GlcNAc (9.6%) and α(1, 6)-linked fucose (32.6%) as complex glycans. In contrast, gp120-HDEL produced in *N. benthamiana* without kifunensine treatment showed increased HMG content (91%) (Man_5−9_GlcNAc_2_) (Figure 4 and Table 1), signifying that the protein was retained in the ER, although there was a small percentage of complex glycans, including GlcNAc-linked and plant-specific β(1, 2)-xylose glycoforms. Thus, it is evident that a minor fraction of gp120-HDEL escaped from the ER into the Golgi apparatus. This in turn highlights the limitation of the H/KDEL signal-based ER-retention strategy to restrict glycosylation heterogeneity, as we have previously shown with gCTB-KDEL (Matoba, 2015). A previous study by Rosenberg et al. showed that the glycan composition of a plant-produced gp140-KDEL had a similar HMG-rich profile, but with no detectable plant-specific glycoforms (Rosenberg et al., 2013). However, this could be due to differences in expression vector/conditions, the envelope glycoproteins used (C clade Du156 gp120 in the present study vs. SHIV-89.6P gp140 in Rosenberg et al.), and/or the methods used for glycan analysis. Meanwhile, for plants treated with kifunensine, the HIV envelope protein showed a distinct HMG-rich glycan profile with a high percentage of Man9 (64.5%), which was much higher than that (20.3%) of the protein produced without kifunensine treatment (Figure 4 and Table 1). Interestingly, there was a small percentage of α(1, 3)-linked glucose structure (11.3%), which may represent incompletely folded or misfolded gp120 (Dejgaard et al., 2004). This might partly explain ER stress and the reduction of production yield upon kifunensine treatment. Since kifunensine is an α-mannosidase I inhibitor that blocks the processing of Man9 HMGs to other glycoforms, the significantly high content of Man9 structure indicates the effectiveness (albeit not perfect) of the present kifunensine treatment conditions for the transient overexpression of Man9-rich glycoproteins in whole plants. In this study, we did not examine the vaccine efficacy of the Man9-rich gp120 expressed under kifunensine-treated conditions, because monomeric gp120 is ineffective at inducing HIV-neutralizing antibodies. Development of a stable and soluble trimeric gp120 vaccine antigen inducing broadly neutralizing antibodies remains to be a major challenge in HIV vaccine research (Karlsson Hedestam et al., 2017; Ward and Wilson, 2017). Nevertheless, the results presented herein provide a basis to perform such a study using trimeric gp120, when it becomes available, as Man9-rich HMG glycans may mimic the glycosylation profile of the natural envelope glycoprotein on HIV virions while enhancing vaccine efficacy via increased affinity to C-type lectin receptors (Doores et al., 2010; Eggink et al., 2010; Bonomelli et al., 2011).

**Table 1:** Relative *N*-glycan composition of gp120 expressed in different growth conditions: HEK produced gp120, *Nicotiana benthamiana* produced gp120-HDEL and *Nicotiana benthamiana produced* gp120-HDEL + 3 Kif.

**Structure**	**Ratio (%)**			
		**HEK_gp120_**	**Gp120-HDEL**	**Gp120-HDEL + 3 Kif**
Mannose-type structure	M3	0.5	–	–
	M4	1.1	1.8	–
	M5	9.2	4.2	1.1
	M6B	4.6	7.2	8.9
	M7A	4.5	5.0	1.2
	M7B	1.0	11.9	1.8
	M8A	10.3	41.1	9.8
	M8B	0.9	–	1.4
	M9	7.3	20.3	64.5
Glc-linked structure	GlcM6	–	1.0	7.8
	GlcM7	–	–	3.5
GlcNAc-linked structure	^GN^M3	–	4.7	–
	_GN_M3	3.2	–	–
	GN2M3	4.9	–	–
	GNM5	0.9	–	–
	GN3M3	0.6	–	–
β 1,2-Xyl-linked structure	GNM3X	–	2.8	–
α 1,6-Fuc-linked structure	^GN^M3F	4.4	–	–
	_GN_M3F	1.3	–	–
	GN2M3F	18.0	–	–
	GN3M3F	7.0	–	–
	GN4M3F	1.9	–	–
β 1,4-Gal-linked structure	GalGN2M3	2.1	–	–
	GalGN2M3F	9.8	–	–
	GalGN3M3F	2.8	–	–
	GalGNM5	0.6	–	–
	Gal2GN2M3F	3.1	–	–
Total Mannose-type structure		39.4	91.5	88.7
Total GlcNAc-linked structure		9.6	4.7	–
Total Fuc-linked structure		32.6	–	–
Total Gal-linked structure		18.4	–	–

To conclude, we have shown that the hydroponic treatment of *N. benthamiana* with kifunensine allows us to obtain Man9-rich HMG-displaying recombinant glycoproteins upon transient overexpression. Our findings warrant further studies evaluating the effectiveness of kifunensine treatment for other glycoproteins, particularly those without a H/KDEL tag, optimization of hydroponic culture conditions, and feasibility of this approach for large-scale production. With additional investigations for glycosylation and bioprocess optimizations, our strategy discussed here opens up new possibilities of producing mannosylated recombinant vaccine antigens that can be efficiently targeted to C-type lectin receptors. Identification and characterization of *N. benthamiana* mannosidase(s) targeted by kifunensine may aid in understanding the glycosylation regulation in plants and developing glyco-engineered host plants for vaccine production.

## Author contributions

NM: Conceived of and designed the study; SR and YO: Performed experiments and contributed equally to the work; HK: Performed glycan analysis; SR, YO, KH, KF, and NM: Analyzed data; SR, YO, and NM: Wrote the manuscript. All authors reviewed the manuscript.

### Conflict of interest statement

The authors declare that the research was conducted in the absence of any commercial or financial relationships that could be construed as a potential conflict of interest.

## References

[B1] Al-Barwani F. Young S. L. Baird M. A. Larsen D. S. Ward V. K. (2014). Mannosylation of virus-like particles enhances internalization by antigen presenting cells. PLoS ONE 9:e104523. 10.1371/journal.pone.0104523 25122183 PMC4133192

[B2] Apostolopoulos V. Thalhammer T. Tzakos A. G. Stojanovska L. (2013). Targeting antigens to dendritic cell receptors for vaccine development. J. Drug Deliv. 2013:869718. 10.1155/2013/869718 24228179 PMC3817681

[B3] Bardor M. Faveeuw C. Fitchette A. C. Gilbert D. Galas L. Trottein F.. (2003). Immunoreactivity in mammals of two typical plant glyco-epitopes, core α(1,3)-fucose and core xylose. Glycobiology 13, 427–434. 10.1093/glycob/cwg024 12626420

[B4] Behrens A. J. Crispin M. (2017). Structural principles controlling HIV envelope glycosylation. Curr. Opin. Struct. Biol. 44, 125–133. 10.1016/j.sbi.2017.03.008 28363124 PMC5513759

[B5] Bonomelli C. Doores K. J. Dunlop D. C. Thaney V. Dwek R. A. Burton D. R. (2011). The glycan shield of HIV is predominantly oligomannose independently of production system or viral clade. PLoS ONE 6:e23521 10.1371/journal.pone.0023521 21858152 PMC3156772

[B6] Chen Q. Lai H. Hurtado J. Stahnke J. Leuzinger K. Dent M. (2013). Agroinfiltration as an effective and scalable strategy of gene delivery for production of pharmaceutical proteins. Adv. Tech. Biol. Med. 1:103. 10.4172/atbm.1000103 25077181 PMC4113218

[B7] Chichester J. A. Manceva S. D. Rhee A. Coffin M. V. Musiychuk K. Mett V.. (2013). A plant-produced protective antigen vaccine confers protection in rabbits against a lethal aerosolized challenge with *Bacillus anthracis* Ames spores. Hum. Vaccin. Immunother. 9, 544–552. 10.4161/hv.23233 23324615 PMC3891710

[B8] D'Aoust M. A. Lavoie P. O. Couture M. M. Trépanier S. Guay J. M. Dargis M.. (2008). Influenza virus-like particles produced by transient expression in *Nicotiana benthamiana* induce a protective immune response against a lethal viral challenge in mice. Plant Biotechnol. J. 6, 930–940. 10.1111/j.1467-7652.2008.00384.x 19076615

[B9] Dejgaard S. Nicolay J. Taheri M. Thomas D. Y. Bergeron J. J. (2004). The ER glycoprotein quality control system. Curr. Issues Mol. Biol. 6, 29–42. 10.21775/cimb.006.029 14632257

[B10] Doores K. J. Bonomelli C. Harvey D. J. Vasiljevic S. Dwek R. A. Burton D. R.. (2010). Envelope glycans of immunodeficiency virions are almost entirely oligomannose antigens. Proc. Natl. Acad. Sci. U.S.A. 107, 13800–13805. 10.1073/pnas.1006498107 20643940 PMC2922250

[B11] Eggink D. Melchers M. Wuhrer M. Van Montfort T. Dey A. K. Naaijkens B. A.. (2010). Lack of complex N-glycans on HIV-1 envelope glycoproteins preserves protein conformation and entry function. Virology 401, 236–247. 10.1016/j.virol.2010.02.019 20304457 PMC3776475

[B12] Elbein A. D. Tropea J. E. Mitchell M. Kaushal G. P. (1990). Kifunensine, a potent inhibitor of the glycoprotein processing mannosidase I. J. Biol. Chem. 265, 15599–15605. 2144287

[B13] Feinberg H. Castelli R. Drickamer K. Seeberger P. H. Weis W. I. (2007). Multiple modes of binding enhance the affinity of DC-SIGN for high mannose N-linked glycans found on viral glycoproteins. J. Biol. Chem. 282, 4202–4209. 10.1074/jbc.M609689200 17150970 PMC2277367

[B14] Feinberg H. Mitchell D. A. Drickamer K. Weis W. I. (2001). Structural basis for selective recognition of oligosaccharides by DC-SIGN and DC-SIGNR. Science 294, 2163–2166. 10.1126/science.1066371 11739956

[B15] Garcia J. P. Beingesser J. Bohorov O. Bohorova N. Goodman C. Kim D.. (2014). Prevention and treatment of *Clostridium perfringens* epsilon toxin intoxication in mice with a neutralizing monoclonal antibody (c4D7) produced in *Nicotiana benthamiana*. Toxicon 88, 93–98. 10.1016/j.toxicon.2014.06.012 24950050 PMC4119486

[B16] Gomord V. Fitchette A. C. Menu-Bouaouiche L. Saint-Jore-Dupas C. Plasson C. Michaud D.. (2010). Plant-specific glycosylation patterns in the context of therapeutic protein production. Plant Biotechnol. J. 8, 564–587. 10.1111/j.1467-7652.2009.00497.x 20233335

[B17] Hamorsky K. T. Grooms-Williams T. W. Husk A. S. Bennett L. J. Palmer K. E. Matoba N. (2013a). Efficient single tobamoviral vector-based bioproduction of broadly neutralizing anti-HIV-1 monoclonal antibody VRC01 in *Nicotiana benthamiana* plants and utility of VRC01 in combination microbicides. Antimicrob. Agents Chemother. 57, 2076–2086. 10.1128/AAC.02588-12 23403432 PMC3632893

[B18] Hamorsky K. T. Kouokam J. C. Bennett L. J. Baldauf K. J. Kajiura H. Fujiyama K.. (2013b). Rapid and scalable plant-based production of a cholera toxin B subunit variant to aid in mass vaccination against cholera outbreaks. PLoS Negl. Trop. Dis. 7:e2046. 10.1371/journal.pntd.0002046 23505583 PMC3591335

[B19] Hamorsky K. T. Kouokam J. C. Jurkiewicz J. M. Nelson B. Moore L. J. Husk A. S.. (2015). N-glycosylation of cholera toxin B subunit in *Nicotiana benthamiana*: impacts on host stress response, production yield and vaccine potential. Sci. Rep. 5:8003. 10.1038/srep08003 25614217 PMC4303877

[B20] Hiatt A. Bohorova N. Bohorov O. Goodman C. Kim D. Pauly M. H.. (2014). Glycan variants of a respiratory syncytial virus antibody with enhanced effector function and *in vivo* efficacy. Proc. Natl. Acad. Sci. U.S.A. 111, 5992–5997. 10.1073/pnas.1402458111 24711420 PMC4000855

[B21] Iwata Y. Koizumi N. (2005). An Arabidopsis transcription factor, AtbZIP60, regulates the endoplasmic reticulum stress response in a manner unique to plants. Proc. Natl. Acad. Sci. U.S.A. 102, 5280–5285. 10.1073/pnas.0408941102 15781873 PMC555978

[B22] Karauzum H. Chen G. Abaandou L. Mahmoudieh M. Boroun A. R. Shulenin S.. (2012). Synthetic human monoclonal antibodies toward staphylococcal enterotoxin B (SEB) protective against toxic shock syndrome. J. Biol. Chem. 287, 25203–25215. 10.1074/jbc.M112.364075 22645125 PMC3408135

[B23] Karlsson Hedestam G. B. Guenaga J. Corcoran M. Wyatt R. T. (2017). Evolution of B cell analysis and Env trimer redesign. Immunol. Rev. 275, 183–202. 10.1111/imr.12515 28133805 PMC5301504

[B24] Kumar H. Kawai T. Akira S. (2009). Pathogen recognition in the innate immune response. Biochem. J. 420, 1–16. 10.1042/BJ20090272 19382893

[B25] Kwong P. D. Wyatt R. Robinson J. Sweet R. W. Sodroski J. Hendrickson W. A. (1998). Structure of an HIV gp120 envelope glycoprotein in complex with the CD4 receptor and a neutralizing human antibody. Nature 393, 648–659. 10.1038/31405 9641677 PMC5629912

[B26] Lageix S. Lanet E. Pouch-Pelissier M. N. Espagnol M. C. Robaglia C. Deragon J. M. (2008). Arabidopsis eIF2 αkinase GCN2 is essential for growth in stress conditions and is activated by wounding. BMC Plant Biol. 8:134 10.1186/1471-2229-8-134 19108716 PMC2639386

[B27] Lai H. Engle M. Fuchs A. Keller T. Johnson S. Gorlatov S.. (2010). Monoclonal antibody produced in plants efficiently treats West Nile virus infection in mice. Proc. Natl. Acad. Sci. U.S.A. 107, 2419–2424. 10.1073/pnas.0914503107 20133644 PMC2823901

[B28] Lai H. He J. Hurtado J. Stahnke J. Fuchs A. Mehlhop E.. (2014). Structural and functional characterization of an anti-West Nile virus monoclonal antibody and its single-chain variant produced in glycoengineered plants. Plant Biotechnol. J. 12, 1098–1107. 10.1111/pbi.12217 24975464 PMC4175135

[B29] Lam J. S. Huang H. Levitz S. M. (2007). Effect of differential N-linked and O-linked mannosylation on recognition of fungal antigens by dendritic cells. PLoS ONE 2:e1009. 10.1371/journal.pone.0001009 17925857 PMC1995759

[B30] Landry N. Ward B. J. Trépanier S. Montomoli E. Dargis M. Lapini G.. (2010). Preclinical and clinical development of plant-made virus-like particle vaccine against avian H5N1 influenza. PLoS ONE 5:e15559. 10.1371/journal.pone.0015559 21203523 PMC3008737

[B31] Liebminger E. Hüttner S. Vavra U. Fischl R. Schoberer J. Grass J.. (2009). Class I alpha-mannosidases are required for N-glycan processing and root development in *Arabidopsis thaliana*. Plant Cell 21, 3850–3867. 10.1105/tpc.109.072363 20023195 PMC2814498

[B32] Liu L. Wise D. R. Diehl J. A. Simon M. C. (2008). Hypoxic reactive oxygen species regulate the integrated stress response and cell survival. J. Biol. Chem. 283, 31153–31162. 10.1074/jbc.M805056200 18768473 PMC2576535

[B33] Loos A. Van Droogenbroeck B. Hillmer S. Grass J. Pabst M. Castilho A.. (2011). Expression of antibody fragments with a controlled N-glycosylation pattern and induction of endoplasmic reticulum-derived vesicles in seeds of Arabidopsis. Plant Physiol. 155, 2036–2048. 10.1104/pp.110.171330 21325568 PMC3091078

[B34] Mardanova E. S. Kotlyarov R. Y. Kuprianov V. V. Stepanova L. A. Tsybalova L. M. Lomonosoff G. P.. (2015). Rapid high-yield expression of a candidate influenza vaccine based on the ectodomain of M2 protein linked to flagellin in plants using viral vectors. BMC Biotechnol. 15:42. 10.1186/s12896-015-0164-6 26022390 PMC4446962

[B35] Marillonnet S. Giritch A. Gils M. Kandzia R. Klimyuk V. Gleba Y. (2004). In planta engineering of viral RNA replicons: efficient assembly by recombination of DNA modules delivered by Agrobacterium. Proc. Natl. Acad. Sci. U.S.A. 101, 6852–6857. 10.1073/pnas.0400149101 15103020 PMC406431

[B36] Martin G. Sun Y. Heyd B. Combes O. Ulmer J. B. Descours A.. (2008). A simple one-step method for the preparation of HIV-1 envelope glycoprotein immunogens based on a CD4 mimic peptide. Virology 381, 241–250. 10.1016/j.virol.2008.08.039 18835005 PMC2645002

[B37] Massa S. Franconi R. Brandi R. Muller A. Mett V. Yusibov V.. (2007). Anti-cancer activity of plant-produced HPV16 E7 vaccine. Vaccine 25, 3018–3021. 10.1016/j.vaccine.2007.01.018 17280752

[B38] Matoba N. Davis K. R. Palmer K. E. (2011). Recombinant protein expression in Nicotiana. Methods Mol. Biol. 701, 199–219. 10.1007/978-1-61737-957-4_11 21181532

[B39] Matoba N. Kajiura H. Cherni I. Doran J. D. Bomsel M. Fujiyama K.. (2009). Biochemical and immunological characterization of the plant-derived candidate human immunodeficiency virus type 1 mucosal vaccine CTB-MPR. Plant Biotechnol. J. 7, 129–145. 10.1111/j.1467-7652.2008.00381.x 19037902

[B40] Matoba N. (2015). N-Glycosylation of cholera toxin B subunit: serendipity for novel plant-made vaccines? Front. Plant Sci. 6:1132. 10.3389/fpls.2015.01132 26732492 PMC4686596

[B41] McElrath M. J. (2017). Adjuvants: tailoring humoral immune responses. Curr. Opin. HIV AIDS 12, 278–284. 10.1097/COH.0000000000000365 28257301 PMC5510533

[B42] Mett V. Chichester J. A. Stewart M. L. Musiychuk K. Bi H. Reifsnyder C. J.. (2011). A non-glycosylated, plant-produced human monoclonal antibody against anthrax protective antigen protects mice and non-human primates from B. *anthracis* spore challenge. Hum. Vaccin. 7(Suppl.), 183–190. 10.4161/hv.7.0.14586 21270531

[B43] Mett V. Lyons J. Musiychuk K. Chichester J. A. Brasil T. Couch R.. (2007). A plant-produced plague vaccine candidate confers protection to monkeys. Vaccine 25, 3014–3017. 10.1016/j.vaccine.2007.01.017 17287055

[B44] Nandi S. Kwong A. T. Holtz B. R. Erwin R. L. Marcel S. McDonald K. A. (2016). Techno-economic analysis of a transient plant-based platform for monoclonal antibody production. MAbs 8, 1456–1466. 10.1080/19420862.2016.1227901 27559626 PMC5098453

[B45] Petruccelli S. Otegui M. S. Lareu F. Tran Dinh O. Fitchette A. C. Circosta A.. (2006). A KDEL-tagged monoclonal antibody is efficiently retained in the endoplasmic reticulum in leaves, but is both partially secreted and sorted to protein storage vacuoles in seeds. Plant Biotechnol. J. 4, 511–527. 10.1111/j.1467-7652.2006.00200.x 17309727

[B46] Petukhova N. V. Gasanova T. V. Stepanova L. A. Rusova O. A. Potapchuk M. V. Korotkov A. V.. (2013). Immunogenicity and protective efficacy of candidate universal influenza A nanovaccines produced in plants by Tobacco mosaic virus-based vectors. Curr. Pharm. Des. 19, 5587–5600. 10.2174/13816128113199990337 23394564

[B47] Pillet S. Racine T. Nfon C. Di Lenardo T. Z. Babiuk S. Ward B. J.. (2015). Plant-derived H7 VLP vaccine elicits protective immune response against H7N9 influenza virus in mice and ferrets. Vaccine 33, 6282–6289. 10.1016/j.vaccine.2015.09.065 26432915

[B48] Qiu X. Wong G. Audet J. Bello A. Fernando L. Alimonti J. B.. (2014). Reversion of advanced Ebola virus disease in nonhuman primates with ZMapp. Nature 514, 47–53. 10.1038/nature13777 25171469 PMC4214273

[B49] Rosenberg Y. Sack M. Montefiori D. Forthal D. Mao L. Hernandez-Abanto S.. (2013). Rapid high-level production of functional HIV broadly neutralizing monoclonal antibodies in transient plant expression systems. PLoS ONE 8:e58724. 10.1371/journal.pone.0058724 23533588 PMC3606348

[B50] Santi L. Giritch A. Roy C. J. Marillonnet S. Klimyuk V. Gleba Y.. (2006). Protection conferred by recombinant *Yersinia pestis* antigens produced by a rapid and highly scalable plant expression system. Proc. Natl. Acad. Sci. U.S.A. 103, 861–866. 10.1073/pnas.0510014103 16410352 PMC1326254

[B51] Schiller J. T. Lowy D. R. (2015). Raising expectations for subunit vaccine. J. Infect. Dis. 211, 1373–1375. 10.1093/infdis/jiu648 25420478 PMC4400525

[B52] Schneider E. G. Nguyen H. T. Lennarz W. J. (1978). The effect of tunicamycin, an inhibitor of protein glycosylation, on embryonic development in the sea urchin. J. Biol. Chem. 253, 2348–2355. 632274

[B53] Sedaghat B. Stephenson R. Toth I. (2014). Targeting the mannose receptor with mannosylated subunit vaccines. Curr. Med. Chem. 21, 3405–3418. 10.2174/0929867321666140826115552 25174924

[B54] Shoji Y. Jones R. M. Mett V. Chichester J. A. Musiychuk K. Sun X.. (2013). A plant-produced H1N1 trimeric hemagglutinin protects mice from a lethal influenza virus challenge. Hum. Vaccin. Immunother. 9, 553–560. 10.4161/hv.23234 23296194 PMC3891711

[B55] Srivastava I. K. Stamatatos L. Legg H. Kan E. Fong A. Coates S. R.. (2002). Purification and characterization of oligomeric envelope glycoprotein from a primary R5 subtype B human immunodeficiency virus. J. Virol. 76, 2835–2847. 10.1128/JVI.76.6.2835-2847.2002 11861851 PMC135955

[B56] Strasser R. (2016). Plant protein glycosylation. Glycobiology 26, 926–939. 10.1093/glycob/cww023 26911286 PMC5045529

[B57] Takeuchi O. Akira S. (2010). Pattern recognition receptors and inflammation. Cell 140, 805–820. 10.1016/j.cell.2010.01.022 20303872

[B58] Triguero A. Cabrera G. Rodríguez M. Soto J. Zamora Y. Pérez M.. (2011). Differential N-glycosylation of a monoclonal antibody expressed in tobacco leaves with and without endoplasmic reticulum retention signal apparently induces similar *in vivo* stability in mice. Plant Biotechnol. J. 9, 1120–1130. 10.1111/j.1467-7652.2011.00638.x 21819534

[B59] Tsekoa T. L. Lotter-Stark T. Buthelezi S. Chakauya E. Stoychev S. H. Sabeta C.. (2016). Efficient *in vitro* and *in vivo* activity of glyco-engineered plant-produced rabies monoclonal antibodies E559 and 62-71-3. PLoS ONE 11:e0159313. 10.1371/journal.pone.0159313 27427976 PMC4948892

[B60] Van Kooyk Y. Unger W. W. Fehres C. M. Kalay H. García-Vallejo J. J. (2013). Glycan-based DC-SIGN targeting vaccines to enhance antigen cross-presentation. Mol. Immunol. 55, 143–145. 10.1016/j.molimm.2012.10.031 23158834

[B61] Van Liempt E. Bank C. M. Mehta P. Garciá-Vallejo J. J. Kawar Z. S. Geyer R.. (2006). Specificity of DC-SIGN for mannose- and fucose-containing glycans. FEBS Lett. 580, 6123–6131. 10.1016/j.febslet.2006.10.009 17055489

[B62] Vartak A. Sucheck S. J. (2016). Recent advances in subunit vaccine carriers. Vaccines 4:E12. 10.3390/vaccines4020012 27104575 PMC4931629

[B63] Wang S. Takahashi H. Kajiura H. Kawakatsu T. Fujiyama K. Takaiwa F. (2013). Transgenic rice seeds accumulating recombinant hypoallergenic birch pollen allergen Bet v 1 generate giant protein bodies. Plant Cell Physiol. 54, 917–933. 10.1093/pcp/pct043 23539245

[B64] Ward A. B. Wilson I. A. (2017). The HIV-1 envelope glycoprotein structure: nailing down a moving target. Immunol. Rev. 275, 21–32. 10.1111/imr.12507 28133813 PMC5300090

[B65] Whaley K. J. Hiatt A. Zeitlin L. (2011). Emerging antibody products and Nicotiana manufacturing. Hum. Vaccin. 7, 349–356. 10.4161/hv.7.3.14266 21358287 PMC3166493

[B66] Wilson I. B. Zeleny R. Kolarich D. Staudacher E. Stroop C. J. Kamerling J. P.. (2001). Analysis of Asn-linked glycans from vegetable foodstuffs: widespread occurrence of Lewis a, core alpha1,3-linked fucose and xylose substitutions. Glycobiology 11, 261–274. 10.1093/glycob/11.4.261 11358875

[B67] Wu X. Yang Z. Y. Li Y. Hogerkorp C. M. Schief W. R. Seaman M. S.. (2010). Rational design of envelope identifies broadly neutralizing human monoclonal antibodies to HIV-1. Science 329, 856–861. 10.1126/science.1187659 20616233 PMC2965066

[B68] Wycoff K. L. Belle A. Deppe D. Schaefer L. Maclean J. M. Haase S.. (2011). Recombinant anthrax toxin receptor-Fc fusion proteins produced in plants protect rabbits against inhalational anthrax. Antimicrob. Agents Chemother. 55, 132–139. 10.1128/AAC.00592-10 20956592 PMC3019684

[B69] Zhou T. Xu L. Dey B. Hessell A. J. Van Ryk D. Xiang S. H.. (2007). Structural definition of a conserved neutralization epitope on HIV-1 gp120. Nature 445, 732–737. 10.1038/nature05580 17301785 PMC2584968

