# Supplementary material for: Hydroponic Treatment of Nicotiana benthamiana with Kifunensine Modifies the N-glycans of Recombinant Glycoprotein Antigens to Predominantly Man9 High-Mannose Type upon Transient Overexpression
Source: Front Plant Sci. 2018 Jan 30;9:62. doi: 10.3389/fpls.2018.00062 (PMC5797603; doi:10.3389/fpls.2018.00062)
Supplement: Supplementary file 1 [file DataSheet1.pdf]

Supplementary Material

Hydroponic treatment of *Nicotiana benthamiana* with kifunensine modifies the *N*-glycans of recombinant glycoprotein antigens to predominantly the high-mannose type upon transient overexpression

Sugata Roychowdhury[1,#], Young Jun Oh[1,#], Hiroyuki Kajiura[2], Krystal Teasley Hamorsky[1,3], Kazuhito Fujiyama[2], Nobuyuki Matoba[1,4,5,*]

[1]*James Graham Brown Cancer Center, University of Louisville School of Medicine, Louisville, KY, USA*

[2]*The International Center for Biotechnology, Osaka University, Osaka, Japan*
[3]*Department of Medicine, University of Louisville School of Medicine, Louisville, KY, USA*
[4]*Center for Predictive Medicine, University of Louisville School of Medicine, Louisville, KY, USA*

[5]*Department of Pharmacology and Toxicology, University of Louisville School of Medicine, Louisville, KY, USA*

*Correspondence: Nobuyuki Matoba; n.matoba@louisville.edu

1. Supplementary Materials and Methods

Vector construction and expression of gp120 in *N. benthamiana*

The magnICON® system (pICH11599, ICON Genetics, Halle / Saale, Germany) was used for the expression of gp120. The vector was constructed using an *env* clone derived from the CCR5-using clade C virus DU156 (Genbank No. DQ411852). The coding sequence for the gp120 along with a C-terminal HHHHHHDEL amino acid sequence tag was codon optimized for *N. benthamiana* and synthesized by Life Technologies Corporation (Carlsbad, CA), which was cloned into pICH11599 vector via Nco I and Sac I restriction sites to generate pNM367.
For vector inoculation into *N. benthamiana*, pNM367 was co-infiltrated with 2 other magnICON® vectors (pICH20155 and pICH14011) using the vacuum infiltration method as described previously (Matoba et al., 2010).

Extraction and purification of gp120

Leaf materials from either no kif-treated or 3 Kif-treated plants were homogenized by a Waring blender in extraction buffer (17 mM sodium phosphate monobasic monohydrate, 59 mM sodium phosphate dibasic heptahydrate, 68 mM sodium chloride, 40 mM ascorbic acid, pH 6.0). The extract was filtered through 4 layers of cheese cloth and one layer of mira cloth, and then centrifuged at 15,000 x g for 15 min at 4°C. The pH of the supernatant was adjusted to pH 7.2 with 10N NaOH followed by a second spin using same conditions as above. The supernatant was filtered with a 0.22 µm filter and kept cold until loaded onto the column.

Immobilized metal affinity chromatography (IMAC) – The clarified leaf extract was purified using Talon Superflow Metal Affinity Resin (Clontech # 635504), packed in an XK 26/20 column to a 25 mL bed volume on an AKTA purifier. The column was equilibrated with 10 column volumes (CV) of IMAC buffer A (17 mM sodium phosphate monobasic

monohydrate, 59 mM sodium phosphate dibasic heptahydrate, 68 mM sodium chloride, pH 7.2) at 10 mL/min. The clarified extract was loaded at a flow rate of 3.0 mL/min followed by a 10 CV wash with IMAC buffer A at 10 mL/min. Protein was eluted using a step gradient of 100% IMAC buffer B (Buffer A + 150 mM Imidazole, pH 7.2) at 10 mL/min. Protein contained in the major peak was collected as one fraction based on absorbance at 280 nm.

Galanthus nivalis resin chromatography – IMAC eluted gp120 was purified using *Galanthus nivalis* resin packed in a gravity flow column to a 2mL bed volume under ice cold conditions and equilibrated with 10 CV of *Galanthus nivalis* buffer A (17 mM sodium phosphate, monobasic, monohydrate, 59 mM sodium phosphate, dibasic, heptahydrate, 68 mM sodium chloride, pH 7.2). The equilibrated resin was added to IMAC eluted gp120 diluted 8X in *Galanthus nivalis* buffer A and mixed for 1 hour at 4°C with occasional gentle stirring. Post incubation, the gp120 bound resin was added to the gravity flow column and flow through was collected. Resin was washed with 10 CV of *Galanthus nivalis* buffer A and eluted using 75 mL of *Galanthus nivalis* buffer B (17 mM sodium phosphate, monobasic, monohydrate, 59 mM sodium phosphate, dibasic, heptahydrate, 68 mM sodium chloride, 500 mM alpha-Methyl-D-mannopyranoside, pH 4.0) while collecting 5 mL fractions. Collected fractions were combined and continued to DEAE chromatography.

DEAE anion exchange resin chromatography – The *Galanthus nivalis* purified gp120 was diluted 1:10 in DEAE buffer A and the pH was adjusted to 8.0 using 10N NaOH. DEAE resin was packed in a XK 16/20 column to a final bed volume of 20mL on an AKTA purifier. Column was equilibrated with 10 CV of DEAE buffer A (20 mM sodium phosphate, monobasic, monohydrate, pH 8.0) at 10 mL/min. The diluted *Galanthus nivalis* eluted gp120 sample was loaded at a flow rate of 5 mL/min followed by a 10 CV wash with DEAE buffer A at 10 mL/min. Elution was carried out using a linear gradient method to 100% DEAE buffer B (20 mM sodium phosphate, monobasic, monohydrate, 1M sodium chloride, pH 8.0) over 10 CV at 10 mL/min. 20 mL fractions were collected and combined for final formulation and verification.

Combined fractions from DEAE purification were diafiltrated into sterile DPBS using Amicon Ultra-15 30,000 MWCO centrifugal devices (Millipore) according to the manufacturer's instructions. The concentration of HIV-1 gp120 (theoretical extinction coefficients at 280 nm of 1.0884 $(mg/ml)^{-1}$ cm^{-1}) was measured using a bicinchonic acid-based assay (BCA assay; Thermo Scientific, Rockford, IL) with HEKgp120 (Immune Technology, # IT-001-RC1p) as a standard. Integrity and purity of protein bands were analyzed using an overloaded Coomassie-stained 12% SDS-PAGE (5 μg protein per lane) using samples heated at 100°C for 10 minutes in a non-reducing sample buffer. The final gp120 product was also validated using anti-gp120 sandwich ELISA to compare the binding activity to that of a commercial recombinant HEKgp120 protein (Immune Technology, # It-001-RC1p).

Gp120-detection ELISA: ELISA plates were coated overnight at 4°C with the human anti-HIV monoclonal antibody VRC01 produced in plants (Hamorsky et al., 2013) at 10 μg/ml with 100 μl per well. Blocking and washing remains same as described before. Detection of gp120 was achieved by using secondary polyclonal HRP-conjugated goat anti-HIV-1 gp120 antibody (Abcam # ab53840) at 1:500 dilutions and TMB followed by absorbance measurement at 450 nm. Recombinant gp120 produced in HEK293T cells (DU156.12, Clade C (Immunetech # It-001-RC1p) were used as an internal control starting at 1 μg/ml followed by 3-fold dilution in PBS, pH 7.2. All reactions were incubated for 1 hour at room temperature with a final volume of

100 µl in each well carried out in duplicates. Binding curves were generated by GraphPad Prism 6 software using nonlinear regression based on the one site, specific binding equation.

2. Supplementary Figures

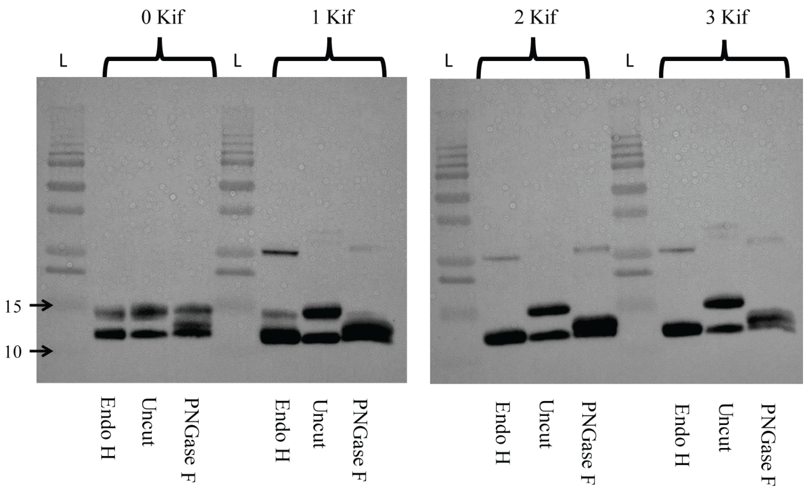

Supplementary Figure 1. Complete immunoblot images for Figure 2A. A representative immunoblot showing gCTB treated with a mock control (uncut), Endo H or PNGase F upon 0, 1, 2 and 3 kif treatments. See the manuscript for details.

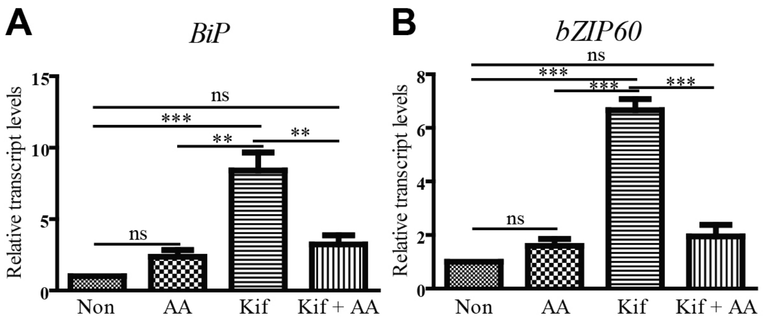

Supplementary Figure 2. qRT-PCR analysis of ER stress related genes in gp120-expressing plants. (A – C) Total leaf RNA was isolated at 2 days post vector inoculation under no kifunensine treatment (Non), no kifunensine treatment with 0.3 mM L-ascorbic acids (Non + AA), 5 mM kinfunensine treatment (3 Kif) or under 5 mM kifunensine plus 0.3 mM L-ascorbic acids (3 Kif + AA). All groups have undergone the same procedural manipulations except for Kif or AA treatments. The expression levels of *BiP* (A), *PDI* (B), and *bZIP60* (C) were quantified by qRT-PCR. The 18S rRNA was used for the normalization of cDNA amount. Values indicated as fold increase to the average normalized value for non-treated plants (Non) and are expressed as means ±SEM of biological replicates (n=9). Statistical significance was analyzed by one-way ANOVA followed by Bonferroni's multiple comparison tests (**$P < 0.01$; ***$P < 0.001$; ns, not significant [$P > 0.05$]).

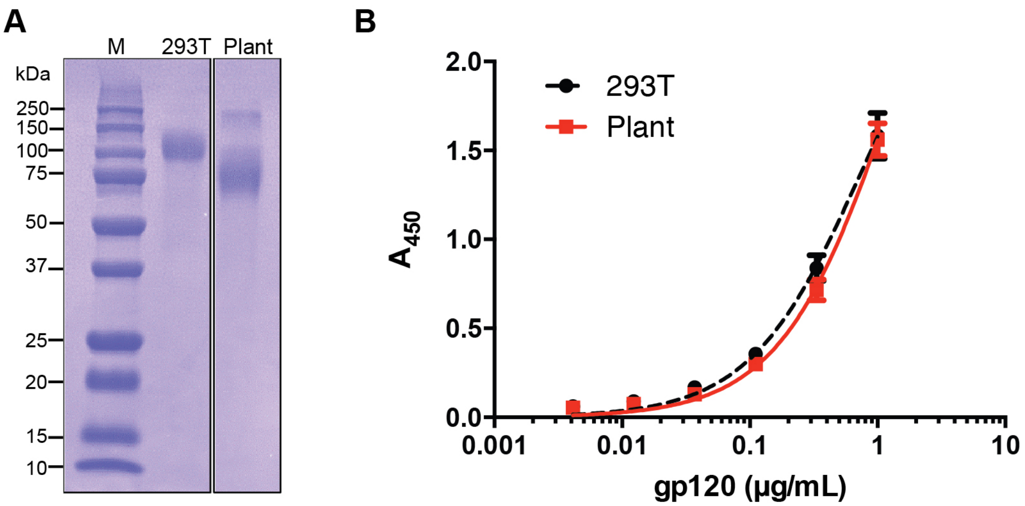

Supplementary Figure 3. Characterization of HIV-1 gp120 produced in *N. benthamiana*. (A) Coomassie-stained 12% SDS-PAGE of gp120 (10 μg) after DEAE purification (M: Marker; 293T: Commercial $gp120_{DU156}$ produced in HEK293T cell line; PLANT: plant-produced recombinant gp120-HDEL). (B) Gp120-detection ELISA verifying the integrity of *N. benthamiana*-produced gp120 (red solid line) compared to a HEK293T cell-produced commercial $gp120_{DU156}$ standard (black dashed line). Binding curves were generated by GraphPad Prism 6 software using nonlinear regression based on the one site, specific binding equation.

References

Hamorsky, K.T., Grooms-Williams, T.W., Husk, A.S., Bennett, L.J., Palmer, K.E., and Matoba, N. (2013). Efficient single tobamoviral vector-based bioproduction of broadly neutralizing anti-HIV-1 monoclonal antibody VRC01 in Nicotiana benthamiana plants and utility of VRC01 in combination microbicides. *Antimicrobial agents and chemotherapy* 57**,** 2076-2086.

Matoba, N., Husk, A.S., Barnett, B.W., Pickel, M.M., Arntzen, C.J., Montefiori, D.C., Takahashi, A., Tanno, K., Omura, S., Cao, H., Mooney, J.P., Hanson, C.V., and Tanaka, H. (2010). HIV-1 neutralization profile and plant-based recombinant expression of actinohivin, an Env glycan-specific lectin devoid of T-cell mitogenic activity. *PLoS ONE* 5**,** e11143.
